# Anatomical Changes after Transcatheter Edge-to-Edge Repair in Functional MR According to MitraClip Generation

**DOI:** 10.3390/jcm12041486

**Published:** 2023-02-13

**Authors:** Alberto Alperi, Pablo Avanzas, Javier Martinez, Antonio Adeba, Iria Silva, Victor Leon, Paula Antuna, Daniel Hernández-Vaquero, Noemi Barja, Félix Fernández, Cesar Moris, Isaac Pascual

**Affiliations:** 1 Hospital Universitario Central de Asturias, 33011 Oviedo, Spain; 2 Instituto de Investigación Sanitaria del Principado de Asturias, ISPA, 33011 Oviedo, Spain; 3 Departamento de Medicina, University of Oviedo, 33003 Oviedo, Spain

**Keywords:** transcatheter mitral edge-to-edge repair, mitral valve, mitral regurgitation

## Abstract

Background: The use of mitral transcatheter edge-to-edge repair (TEER) is rapidly increasing. Anatomical changes have been described after TEER with the MitraClip system in patients with functional mitral regurgitation (MR), although no study has yet evaluated such anatomical impacts in patients treated with the G4 MitraClip generation. Methods: This research constituted a prospective, single-center, observational study including consecutive patients with functional MR. Mitral three-dimensional images were obtained transesophageally with echocardiography before and immediately after TEER. Patients receiving the late-generation (G4) system were compared to those receiving early-generation systems. Results: A total of 116 functional MR patients were evaluated, and 40 (34.5%) and 76 (65.5%) received a late-generation (G4) or early-generation device system, respectively. The baseline clinical and echocardiographic features were well-balanced between the groups. Overall, there was a significant reduction in mitral annular size after the intervention, and greater reductions in the anteroposterior diameter (4 mm vs. 3.54 mm, *p* = 0.03), annular perimeter (11.07 mm vs. 5.29 mm for 3D-perimeter, *p* = 0.001), and annular area (1.29 cm^2^ vs. 1.03 cm^2^, *p* = 0.002) were found for patients receiving the late G4 device generation compared to the early-generation systems. Conclusions: In patients with functional MR, we observed significant changes in mitral valve anatomy with a reduction in anteroposterior diameter, valve perimeter, and area. In our cohort, the extent of those changes was greater with the use of the new-generation G4 MitraClip system compared to prior device generations.

## 1. Introduction

Mitral regurgitation (MR) is the most prevalent heart valve disease in the United States and the second most prevalent in Europe [1]. When mitral valve insufficiency is caused by left ventricular or atrial dilation and dysfunction, leading to impaired leaflet coaptation, it is known as functional (or secondary) MR. No clinical trials have demonstrated an improvement in clinical outcomes for patients with severe functional MR undergoing isolated mitral surgical treatment [2]. However, a randomized clinical trial has demonstrated that a percutaneous approach using the transcatheter edge-to-edge mitral repair (TEER) technique was associated with lower rates of mortality and hospitalization due to heart failure compared to the optimal medical therapy for patients with significant functional MR [3]. Therefore, over the last few years, exponential growth in the use of TEER has been observed, with more than 10,000 cases performed in the United States in the year 2019 [4]. Both the availability of robust clinical data and the appearance of device system iterations are responsible for fueling this transcatheter therapy. More precisely, the later generation of the MitraClip (Abbott Vascular, Menlo Park, CA, USA) system, also called G4, provides a series of novel advantages such as a greater variety of device sizes (4 mm and 6 mm in arm width; 9 mm and 12 mm in arm length; and their combinations) and the possibility of independent leaflet grasping.

Prior studies have reported the anatomical changes that took place at the mitral site after mitral TEER for functional MR, demonstrating a significant reduction in diameter (mainly anteroposterior), perimeter, and area [5,6]. However, no study has yet evaluated the magnitude of those changes in patients treated solely with the G4 device generation. Moreover, the anatomical impact of TEER according to the use of the G4 or previous generations has not been studied either.

Hence, the aim of our study is to analyze the morphological impact of TEER on mitral valve anatomy in patients with severe symptomatic functional MR according to the use of early- or late-generation MitraClip systems.

## 2. Materials and Methods

This research constitutes a prospective, single-center, observational study including consecutive patients with functional moderate-to-severe (3+) or severe (4+) MR who underwent TEER in a tertiary center between 2015 and September 2022. Data regarding previous medical history, procedural details, and clinical outcomes were recorded prospectively in a dedicated database. The decision to undergo percutaneous mitral valve repair was taken individually after comprehensive discussion by a Heart Team. The local ethics committee of the center approved data collection and reporting.

All procedures were performed under general anesthesia with the use of fluoroscopic and transesophageal echocardiographic guidance. Briefly, after atrial transseptal puncture, a guiding catheter was placed within the left atrium across the interatrial septum. The device was then steered and aligned over the origin of the regurgitant jet. Then, the mitral leaflets were grasped upon the advancement of the device into the left ventricle and its subsequent retrieval. Finally, the device was closed, and the mitral leaflets were approximated.

The MitraClip system was used for all cases, and the decision on the number of clips to be implanted was left to the discretion of the interventional team based on residual MR, residual mitral valve area, and diastolic mitral gradients.

### 2.1. Echocardiographic Study

The severity and the etiology of MR was assessed using echocardiography before TEER in accordance with current guidelines [7,8] and classified using a 4-grade scale as in prior TEER trials [3,9]. Residual MR after procedure’s completion was carried out as recommended by current consensus [10]. All patients underwent two- and three-dimensional (3D) transesophageal echocardiography before and immediately after TEER using the EPIQ 7 ultrasound system (Philips; Amsterdam, The Netherlands). For mitral valve anatomic evaluation, three-dimensional images (Zoom 3D, Philips; Amsterdam, The Netherlands) were carefully taken and they were postprocessed with the mitral valve quantification software MVQ QLAB 10.0 (Philips; Amsterdam, The Netherlands). Similar afterload and hemodynamic conditions were ensured before image acquisition for both phases (before and after device’s implantation). Patients with a history of prior mitral annuloplasty and those without 3D images or with 3D images of poor quality (such that adequate mitral valve reconstruction was prevented) were excluded.

### 2.2. Study Outcomes

The main study outcomes were the mitral valve anatomical changes observed after TEER according to the use of an early- or new-generation device. The following anatomical variables were evaluated: anteroposterior diameter, intercommissural diameter, two- and three-dimensional perimeter, and two- and three-dimensional area.

Secondary outcomes were the rates of procedural-related complications and the rates of residual significant (grades 3+ or 4+) MR despite TEER both post-procedure and at 1-year follow-up.

### 2.3. Statistical Analysis

Continuous variables were presented as mean ± standard deviation or median (interquartile range (IQR)), while categorical variables were presented as absolute numbers and percentages. Comparisons were performed using the Student’s *t*-test for normally distributed continuous variables, and the Mann–Whitney U test was used for continuous non-normally distributed variables. Normality was assessed with the Shapiro–Wilk test. The chi square and Fisher’s exact tests were used to compare categorical variables when appropriate. Paired *t*-test was used to compare values between pre- and post-intervention periods.

Two groups were formed for comparison: those patients who received early-generation devices (1st, 2nd, or 3rd generation) and those in whom a new-generation (G4) system was implanted.

A *p* value < 0.05 was considered significant for all statistical tests. All analyses were performed using STATA version 14.0 software (STATA Corp., College Station, TX, USA).

## 3. Results

A total of 171 TEER procedures were performed over the study period, and 119 (69.6%) of them were performed on patients exhibiting functional MR. Three patients were further excluded due to the lack of data regarding mitral annular anatomy (one patient with a prior mitral annuloplasty and two patients with no 3D images that would enable data processing). Therefore, 116 functional MR patients were finally evaluated. Among them, 40 (34.5%) received a late-generation (G4) device system, whereas 76 (65.5%) received prior-generation systems. Baseline clinical characteristics for the overall population of patients with functional MR and according to the generation of the device implanted are displayed in Table 1. All baseline clinical characteristics were well balanced between the study groups. Briefly, the mean age was 73.8 ± 8.8 years and 41 (35.4%) of the patients were female. A substantial number of patients (*n* = 41, 35.4%) exhibited an advanced functional class (NYHA 4) at the time of the procedure, and the mean STS risk score was 4.81 ± 3.1.

The baseline echocardiographic characteristics are displayed in Table 2. The mean left ventricular ejection fraction (LVEF) was 41.4 ± 13%, with no significant differences between device generations (41.1 ± 12.9% for early-generation patients vs. 41.9 ± 14.3% for patients receiving a G4 device, *p* = 0.77). Right ventricular function (tricuspid annular plane systolic excursion (TAPSE) 18 ± 3.6 vs. 18.1 ± 4.2, *p* = 0.92) and estimated systolic pulmonary pressure (46.2 ± 14.4 mmHg vs. 48.6 ± 14.8 mmHg, *p* = 0.47) were also comparable between the study groups. There was a trend towards a higher rate of more severe MR (grade 4+) among the patients receiving late-generation devices (90% vs. 76.3%, *p* = 0.09), which translates to a higher proportion of patients with systolic flow reversal in the left pulmonary veins among the G4 patients (78.9% vs. 47.4%, *p* = 0.05). There were no differences between the study groups with respect to baseline mitral valve anatomic characteristics such as diameters, annular perimeter, annular area, angulation between annular plane and mitral leaflets, or leaflets’ length (Table 2).

The intra-procedural features are summarized in Table 3. A total of 1.47 ± 0.5 and 1.57 ± 0.6 devices were implanted for the early-generation and G4 study groups, respectively. Most cases (99.1%) were performed through the right femoral vein. There was a higher rate of urgent and emergent cases in the G4 study group (32.5% urgent and 15% emergent) compared to the early-generation group (17.1% urgent and 9.2% emergent, *p* = 0.03).

**Figure 1:**
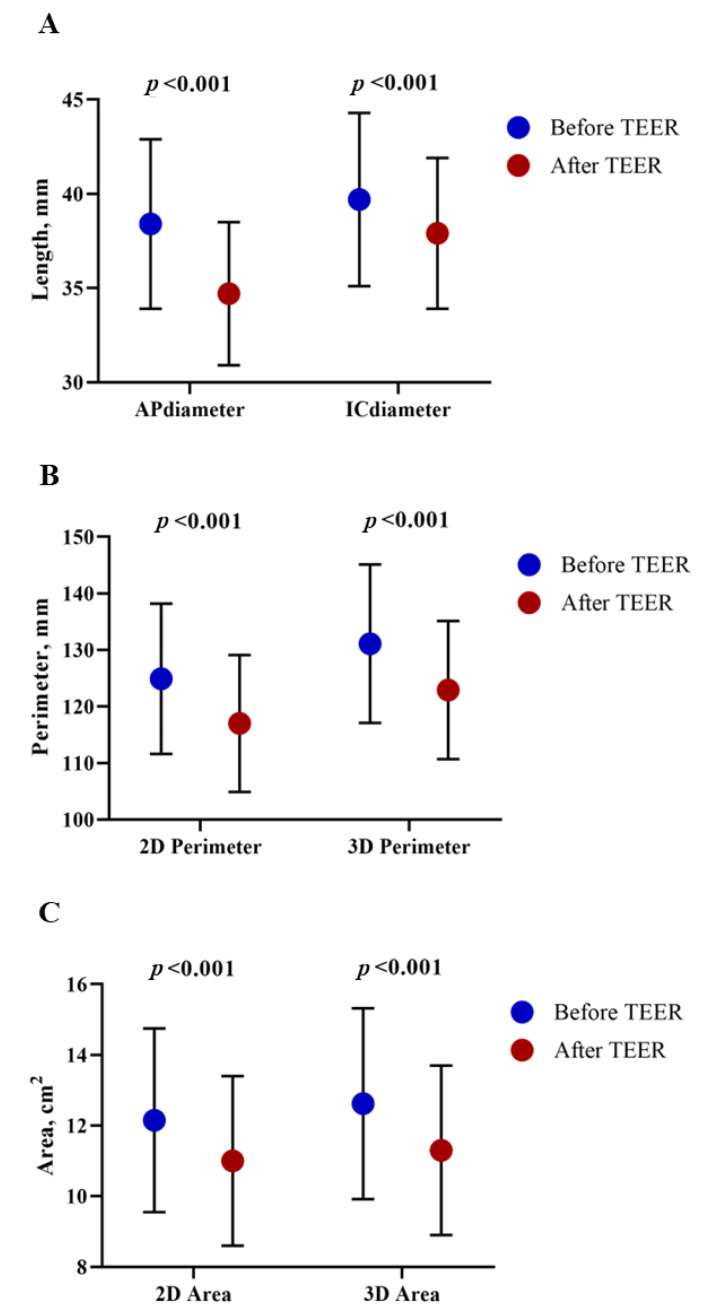
Mean values of mitral valve diameters (**A**), perimeters (**B**), and areas (**C**) before (blue dots) and after (red dots) TEER. Error bars represent standard deviation.

Statistically significant changes in terms of mitral valve geometry were observed after the intervention (Figure 1), with an important reduction in anteroposterior diameter (38.4 ± 4.5 pre-TEER vs. 34.7 ± 3.8 post-TEER, *p* < 0.01), a slight reduction in intercommissural diameter (39.7 ± 4.6 mm pre-TEER vs. 37.9 ± 4 mm post-TEER, *p* < 0.01), and significant reductions in bidimensional and 3D perimeter (131.1 ± 14 mm vs. 122.9 ± 12.2 mm, *p* < 0.001) and area (12.6 ± 2.7 cm^2^ pre-TEER vs. 11.3 ± 2.4 cm^2^ post-TEER, *p* < 0.001).

**Figure 2:**
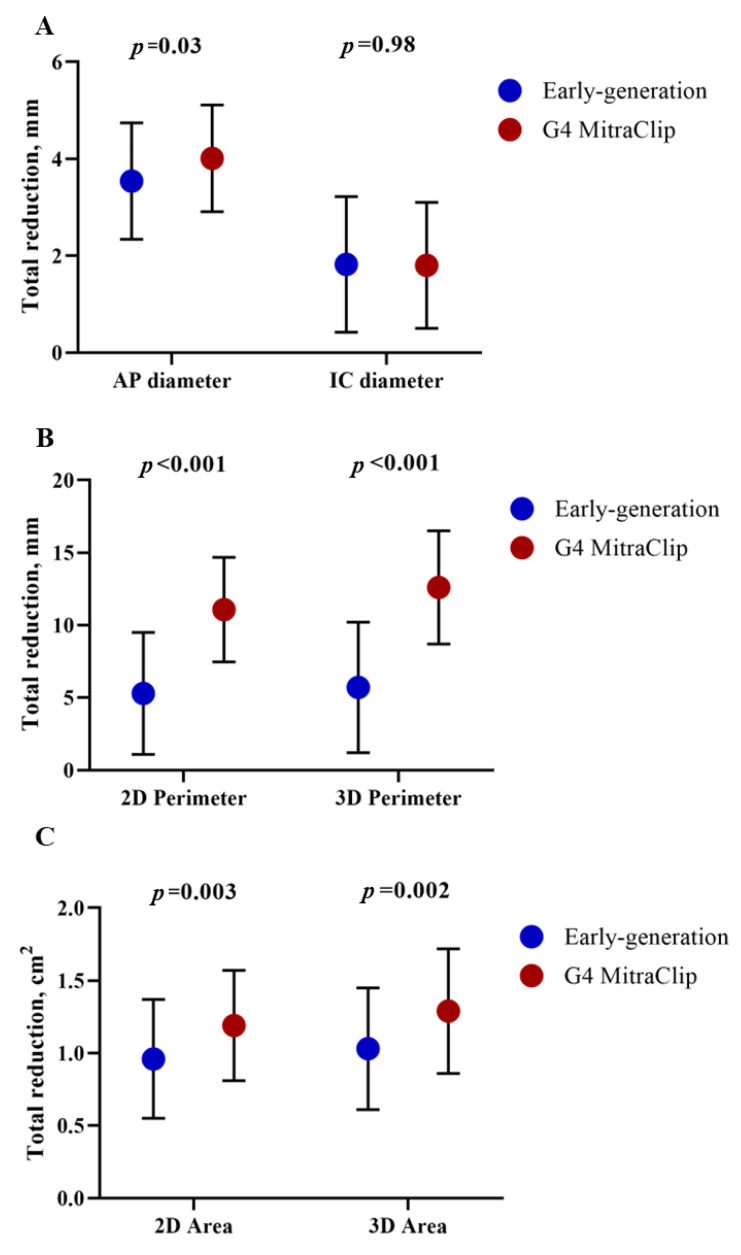
Delta change of mitral valve diameters (**A**), perimeters (**B**), and areas (**C**) after TEER compared to baseline according to the use of early-generation (blue dots) or 4th-generation (red dots) device systems. Error bars represent standard deviation.

When the anatomical changes of the mitral valve were compared according to the device generation employed (Table 4 and Figure 2), greater reductions in the anteroposterior diameter (4 mm vs. 3.54 mm, *p* = 0.03), annular perimeter (11.07 mm vs. 5.29 mm for 3D-perimeter, *p* = 0.001), and annular area (1.29 cm^2^ vs. 1.03 cm^2^, *p* = 0.002) were found for the patients receiving the late G4 device generation, whereas no significant differences were observed regarding the intercommissural diameter (1.82 mm vs. 1.81 mm, *p* = 0.98).

The rates of procedural and in-hospital complications are shown in Table 3 and Table 5. There were two cases of pericardial bleeding leading to pericardiocentesis (one per study group, *p* = 0.98) and two cases of early single-leaflet device attachment (one per study group, *p* = 0.98). No cases of procedural stroke or embolization were observed. There was one in-hospital death per study group (1.32% early-generation vs. 2.5% G4 generation, *p* = 0.98), and no cases of major vascular complications were observed.

The post-procedural MR grade data are shown in Table 3. There were no statistically significant changes between the device generations in terms of residual significant MR (grades 3+ or 4+) after the procedure.

Totals of 68 and 31 patients in the early-generation and G4 groups reached the 1-year follow-up, and, among them, 62 and 29 patients presented effective repairment with residual MR ≤ 2+ immediately after the procedure. Of those patients, 8/62 (12.9%) had MR grade 3+ or 4+ at the 1-year follow-up in the early-generation group, whereas none of the patients within the G4 group exhibited such a recurrence in significant MR (*p* = 0.05).

## 4. Discussion

The main findings of our study can be summarized as follows: (i) significant changes in mitral valve anatomy exist after mitral TEER among patients with functional MR; (ii) in our cohort, the quantities of these changes were different based on the generation of MitraClip device used, with the G4 generation system exhibiting greater reductions in anteroposterior diameter, valve perimeter, and area; and (iii) the rates for procedural related complications were very low for both the early-generation and new-generation systems.

Experience is growing rapidly in the TEER field, with a progressive annual increase in terms of procedural volume. Open-heart surgery is not frequently performed for patients with isolated functional MR, and a randomized clinical trial has demonstrated the superiority of TEER when added to an optimal medical therapy compared to medical therapy alone [3]. Moreover, medical therapy directed towards heart failure patients has not been demonstrated to systematically reduce functional MR. A recent randomized trial reported a reduction in MR severity with the use of sacubitril/valsartan, but the vast majority of patients in that study presented moderate or mild MR; thus, this trial’s results may not apply to patients with MR grades 3+ and 4+ [11]. Therefore, a higher number of patients are expected to undergo TEER over the upcoming years, and a comprehensive analysis of the changes that take place during this intervention seems mandatory.

Mitral valve anatomical changes in functional MR: Late iterations in image acquisition and software quantification enable a comprehensive echocardiographic assessment of the mitral valve complex. There is growing interest in the thorough anatomical study of the mitral valve regarding patients undergoing transcatheter interventions. Prior studies with early-generation systems demonstrated a reduction in mitral annular dimensions after TEER in patients with functional MR [5,12,13]. Additionally, other investigations have demonstrated a significant association between the reduction in the anteroposterior diameter [14] and the ratio between the total leaflet area and total annular area [15] with residual MR. To the best of our knowledge, this is the first study to compare, from a comprehensive 3D anatomic standpoint, the differences that take place during TEER between the current and prior-generation systems. The fact that the anteroposterior diameter and annular perimeters and areas were more significantly decreased with the G4 systems might be linked to the greater grasping of leaflets’ tissue due to the longer and wider arms available. Increased operator experience and improved patient selection based on pre-operative echo data could also have played a role in these findings, although the plausibility for a real difference driven by the device generation is high. Our results should be further supported in larger cohorts, but they still have significant clinical value in their current form, as greater reductions in annular dimensions may not only be associated with a greater acute decline in MR, but also with a lower recurrence of significant MR during follow-up. Future studies are warranted.

Tailored device selection: The G4 MitraClip increases the variety of sizes compared to its predecessors (Classic, NT, NTR, and XTR) as it offers four different clips: the NT and XT (similar in size to NTR and XTR) and the NTW and XTW, which have 50% wider arms. Hence, nowadays, tailored device selection for every specific type of anatomy should be carefully considered. This new generation with the availability of larger and wider arms facilitates leaflet tissue insertion in the grasping area, which can be of upmost importance in certain clinical settings such as in wide coaptation gaps and severely dilated mitral annuli [16]. This expanded armamentarium of devices gives physicians the ability to choose a clip size or combination of clips based on each patient’s mitral valve anatomy. Moreover, the two grippers on the clip arms of the MitraClip system did not move independently in the initial generations, while with the G4 it is possible to grasp the anterior and posterior mitral leaflets separately. This tool might be highly useful for restoring leaflet coaptation when marked posterior tethering and large coaptation gaps are present.

Procedural safety and outcomes: Since the emergence of mitral TEER, procedural safety has been one of its main strengths. The rates of serious complications within 30 days after a procedure have always been low and have further decreased over time. These promising results are likely due to a combination of factors, including growing operator experience and improved patient selection. Notably, neither cases of major vascular access complications nor stroke events were observed in our series, and the rates of pericardial bleeding (<2%) and minor vascular complications (<3%) were relatively low. Moreover, it is worth mentioning that a growing number of cases were performed urgently or emergently over time, with no apparent trade-off in terms of procedural safety.

In the G4 subgroup, 90% of the patients exhibited MR ≤ 2+ after device implantation, with > 70% of them showing MR 1+. These results did not reach statistical significance when compared to the early-generation cases. A limitation in statistical power due to both the sample size and the number of cases with residual significant MR may have also accounted for these results. However, we have observed a trend towards a greater durability of the mitral repairment (when this was initially effective) with the G4 generation compared to the early-generation group, with no recurrences of significant MR at 1-year follow-up in the former vs. 12.9% in the latter. Future studies with greater statistical power should help underscore whether the G4 generation provides superior results in terms of early residual MR and hard clinical endpoints (e.g., hospitalization due to heart failure, mortality, etc.) compared to older device generations.

### Limitations

Our study has some limitations. First, its observational nature might have led to unknown, unbalanced characteristics between the study groups. Moreover, patient selection bias should be taken into account despite the apparent good comparability between the study groups, as the group allocation procedure employed did not exploit a randomization process. Second, no core lab was used for the assessment of the echocardiographic parameters. However, all studies were read by the same two experienced operators in TEER and mitral valve disease, hence reducing the possibility for biased results. Third, this was a single-center study, and the generalizability of these findings must be proven using larger and multi-center cohorts. Finally, longer-term follow-ups and new studies are warranted to provide more insight into the durability of the repair and relevant clinical outcomes such as heart failure readmission and mortality according to the device generation used.

## 5. Conclusions

In this series of patients with functional MR, we observed significant changes in mitral valve anatomy after mitral TEER with a reduction in anteroposterior diameter, valve perimeter, and area. In our cohort, the extent of these changes was greater with the use of the new-generation G4 MitraClip system compared to prior device generations.

## Figures and Tables

**Table 1:** Baseline clinical characteristics of patients with functional MR according to the implantation of early-generation vs. 4th-generation MitraClip system.

	Overall (*n* = 116)	Early-Generation (*n* = 76)	4th-Generation (*n* = 40)	*p* Value
Age, years	73.8 ± 8.8	74.5 ± 7.9	72.8 ± 10.3	0.32
Female	41 (35.4)	25 (32.9)	16 (40)	0.45
Body surface area	1.84 ± 0.22	1.82 ± 0.22	1.86 ± 0.23	0.33
Hypertension	85 (73.2)	58 (76.3)	27 (67.5)	0.31
Diabetes	46 (39.7)	31 (40.8)	15 (37.5)	0.73
Dyslipidemia	69 (59.5)	42 (55.3)	27 (67.5)	0.21
Chronic kidney disease	67 (57.8)	42 (55.3)	25 (62.5)	0.45
Peripheral artery disease	21 (18.1)	13 (17.1)	8 (20)	0.71
Chronic obstructive pulmonary disease	27 (23.3)	19 (25)	8 (20)	0.55
Malignancy		5 (6.6)	2 (5)	0.74
PCI	45 (38.8)	28 (36.8)	17 (42.5)	0.55
CABG	13 (11.3)	9 (12)	4 (10)	0.75
Atrial fibrillation	71 (61.2)	47 (61.8)	24 (60)	0.85
Prior aortic valve replacement	1 (0.9)	1 (1.3)	0	0.46
Pacemaker	10 (8.6)	7 (9.2)	3 (7.5)	0.76
ICD	27 (23.3)	19 (25)	8 (20)	0.55
Cardiac resynchronization therapy	13 (11.2)	10 (13.2)	3 (7.5)	0.35
Creatinine, mg/dl	1.5 ± 0.8	1.49 ± 1.1	1.52 ± 0.67	0.85
Hemoglobin, g/dl	11.94 ± 1.8	11.97 ± 1.9	11.91 ± 1.6	0.86
NYHA I-III	75 (64.6)	51 (77.1)	25 (62.5)	0.62
NYHA IV	41 (35.4)	25 (32.9)	15 (37.5)	
STS-PROM	4.3 (2.61–2.16)	4.2 (2.7–6.7)	4.6 (2.3–5.8)	0.85
Baseline medical treatment				
ARNI/RASI	74 (63.8)	50 (65.8)	24 (60)	0.54
MRA	8 (7)	6 (8)	2 (5)	0.55
Beta-blockers	97 (84.4)	64 (85.3)	33 (82.5)	0.69
Loop diuretics	107 (93)	72 (96)	35 (87.5)	0.11
OAC	73 (62.9)	47 (61.8)	26 (65)	0.74

Data are expressed as mean ± SD, median (interquartile range), or *n* (%). ARNI: Angiotensin receptor neprilysin inhibitor. CABG: coronary artery bypass graft. ICD: implantable cardioverter defibrillator. MRA: mineralocorticoid receptor antagonist. NYHA: New York Heart Association. OAC: oral anticoagulant. PCI: percutaneous coronary intervention. RASI: renin–angiotensin system inhibitors. STS-PROM: society of thoracic surgeons predictive risk of mortality.

**Table 2:** Baseline echocardiographic characteristics.

	Overall (*n* = 116)	Early-Generation (*n* = 76)	4th-Generation (*n* = 40)	*p* Value
LVEF, %	41.4 ± 13	41.1 ± 12.9	41.9 ± 14.3	0.77
iTDLVV, mL/m^2^	79 ± 29	82.1 ± 31.1	72 ± 25	0.11
iTDLVD, mm/m^2^	31.9 ± 6.9	32.4 ± 7.1	30.6 ± 6.3	0.19
Left atrial volume, mL	108.8 ± 42	108.2 ± 50	110.8 ± 36	0.83
TAPSE, mm	18 ± 3.8	18 ± 3.6	18.1 ± 4.2	0.92
RVTDD, mm	42.5 ± 6.4	42.4 ± 5.9	42.9 ± 7.3	0.74
PASP, mmHg	47.1 ± 14.5	46.2 ± 14.4	48.6 ± 14.8	0.47
MR grade,				0.09
Grade 3+	22 (19)	18 (23.7)	4 (10)	
Grade 4+	94 (81)	58 (76.3)	36 (90)	
ERO, cm^2^	0.39 ± 0.12	0.39 ± 0.10	0.39 ± 0.17	0.77
Systolic reversal pulmonary vein flow (*n* = 76)	42 (55.3)	27 (47.4)	15 (78.9)	0.03
Mitral valve anatomical characteristics				
Anteroposterior diameter, mm	38.4 ± 4.5	38.3 ± 4.9	38.8 ± 3.7	0.53
Intercommissural diameter, mm	39.7 ± 4.6	39.44 ± 4.72	40.41 ± 4.33	0.27
2D annular perimeter, mm	124.9 ± 13.3	124.4 ± 13.4	126.1 ± 13.3	0.50
3D annular perimeter, mm	131.1 ± 14	130.3 ± 14.4	132.5 ± 13.2	0.43
2D annular area, cm^2^	12.15 ± 2.7	12.01 ± 2.7	12.4 ± 2.6	0.43
3D annular area, cm^2^	12.62 ± 2.8	12.49 ± 2.8	12.88 ± 2.7	0.46
Anterior leaflet angulation, °	26.8 ± 5.6	27.1 ± 6.1	25.7 ± 3.5	0.18
Posterior leaflet angulation, °	40.3 ± 8.7	40.7 ± 9.8	39.4 ± 5.2	0.49
Anterior leaflet length, mm	26.1 ± 3.2	25.81 ± 3.1	26.4 ± 3.4	0.34
Posterior leaflet length, mm	13.22 ± 2.5	13.4 ± 2.42	12.9 ± 2.83	0.32

Data are expressed as *n* (%) or mean ± SD. 2D: 2 dimensions. 3D: 3 dimensions. ERO: Effective regurgitant orifice. iTDLVD: index-telediastolic left ventricular diameter. iTDLVV: index-telediastolic left ventricular volume antero-posterior. LVEF: left ventricular ejection fraction. MR: mitral regurgitation. PASP: pulmonary artery systolic pressure. RVTDD: right ventricle telediastolic diameter. TAPSE: tricuspid annular plane systolic excursion. °: degrees.

**Table 3:** Procedural features for the whole cohort and according to the device generation used.

	Overall (*n* = 116)	Early-Generation (*n* = 76)	4th-Generation (*n* = 40)	*p* Value
Numbers of Clips implanted	1.51 ± 0.5	1.47 ± 0.5	1.57 ± 0.6	0.37
Types of Clips				
1st Generation		18 (23.7)		
2nd Generation		17 (22.4)		
3rd Generation				
NTr		8 (10.5)		
XTr		33 (43.4)		
4th Generation				
NT			1 (2.5)	
NTw			13 (32.5)	
XT			3 (7.5)	
XTw			23 (57.5)	
Vascular access				0.35
Right femoral	115 (99.1)	76 (100)	39 (97.5)	
Left femoral	1 (0.9)		1 (2.5)	
Timing of intervention				0.04
Elective	77 (66.4)	56 (73.7)	21 (52.5)	
Urgent	26 (22.4)	13 (17.1)	13 (32.5)	
Emergent	13 (11.2)	7 (9.2)	6 (15)	
Procedural complications				
Tamponade	2 (1.7)	1 (1.3)	1 (2.5)	0.98
Stroke	0	0	0	NA
SLDA	2 (1.7)	1 (1.3)	1 (2.5)	0.98
Embolization	0	0	0	NA
Technical success	113 (97.4)	74 (97.3)	39 (97.5)	0.99
MR after intervention				0.65
1+	84 (72.41)	55 (72.36)	29 (72.5)	
2+	20 (17.24)	14 (18.42)	7 (17.5)	
3+	10 (8.62)	6 (7.89)	3 (7.5)	
4+	2 (1.72)	1 (1.32)	1 (2.5)	
Mean diastolic gradient, mmHg	3.45 ± 1.3	3.43 ± 1.3	3.50 ± 1.5	0.79

Data are expressed as mean ± SD or *n* (%). MR: mitral regurgitation. SLDA: single-leaflet device attachment.

**Table 4:** Absolute reductions in mitral valve anatomic parameters according to device generation (1st, 2nd, and 3rd generation vs. 4th generation).

	Early Generation (*n* = 76)	4th Generation (*n* = 40)	Difference †	*p* Value
δAnteroposterior diameter, mm	3.54 ± 1.2	4.01 ± 1.1	0.47	0.03
δIntercommissural diameter, mm	1.82 ± 1.4	1.81 ± 1.3	0.01	0.98
δ2D annular perimeter, mm	5.29 ± 4.2	11.07 ± 3.6	5.8	0.001
δ3D annular perimeter, mm	5.70 ± 4.5	12.61 ± 3.9	6.9	0.001
δ2D annular area, cm^2^	0.96 ± 0.41	1.19 ± 0.38	0.22	0.003
δ3D annular area, cm^2^	1.03 ± 0.42	1.29 ± 0.43	0.26	0.002

† The difference between groups is displayed as 4th Generation versus Early generation. δ expresses the absolute reduction in this parameter between pre- and post-TEER. Data are expressed as mean ± SD.

**Table 5:** In-hospital outcomes for the whole cohort and according to the device generation used.

	Overall (*n* = 116)	Early Generation (*n* = 76)	4th Generation (*n* = 40)	*p* Value
All-cause mortality	2 (1.72)	1 (1.32)	1 (2.5)	0.64
Stroke	0	0	0	NA
Major vascular complication	0	0	0	NA
Minor vascular complication	3 (2.59)	2 (2.63)	1 (2.5)	0.97
Major bleeding	2 (1.72)	1 (1.32)	1 (2.5)	0.64
AKI stages 2 or 3	8 (6.9)	5 (6.6)	3 (7.5)	0.86

Data are expressed as mean ± SD or *n* (%). AKI: acute kidney injury.

## Data Availability

The data that support the findings of this study are available from the corresponding author on reasonable request.
